# Glycolysis-related biomarker TCIRG1 participates in regulation of renal cell carcinoma progression and tumor immune microenvironment by affecting aerobic glycolysis and AKT/mTOR signaling pathway

**DOI:** 10.1186/s12935-023-03019-0

**Published:** 2023-08-30

**Authors:** Sichen Di, Min Gong, Jianmin Lv, Qiwei Yang, Ye Sun, Yijun Tian, Cheng Qian, Wenjin Chen, Wang Zhou, Keqin Dong, Xiaokai Shi, Yuning Wang, Hongru Wang, Jian Chu, Sishun Gan, Xiuwu Pan, Xingang Cui

**Affiliations:** 1 Department of Urinary Surgery, Postgraduate Training Base at Shanghai Gongli Hospital, Ningxia Medical University, Yinchuan, Ningxia China; 2 Department of Urology, Xinhua Hospital, School of Medicine, Shanghai Jiaotong University, 1665 Kongjiang Road, Shanghai, 200092 China; 3 Department of Urology, Third Affiliated Hospital of the Second Military Medical University, Shanghai, 201805 China; 4 Department of Urology, Seventh People’s Hospital of Shanghai University of Traditional Chinese Medicine, Shanghai, 200137 China; 5 Department of Urology, Shanghai East Hospital, School of Medicine, Tongji University, Shanghai, 200100 China; 6 Department of Urology, The Affiliated Changzhou Second People’s Hospital of Nanjing Medical University, Changzhou, 213000 China; 7 Department of Urinary Surgery, Gongli Hospital, Second Military Medical University (Naval Medical University), Shanghai, China; 8 Department of Urology, Shanghai Baoshan Luodian Hospital, Shanghai, 201908 China

**Keywords:** Clear cell renal cell carcinoma, Tumor immune microenvironment, Aerobic glycolysis, TCIRG1, Biomarker

## Abstract

**Background:**

Renal cell carcinoma (RCC) is a hypermetabolic disease. Abnormal up-regulation of glycolytic signaling promotes tumor growth, and glycolytic metabolism is closely related to immunotherapy of renal cancer. The aim of the present study was to determine whether and how the glycolysis-related biomarker TCIRG1 affects aerobic glycolysis, the tumor microenvironment (TME) and malignant progression of clear cell renal cell carcinoma (ccRCC).

**Methods:**

Based on The Cancer Genome Atlas (TCGA, n = 533) and the glycolysis-related gene set from MSigDB, we identified the glycolysis-related gene TCIRG1 by bioinformatics analysis, analyzed its immunological properties in ccRCC and observed how it affected the biological function and glycolytic metabolism using online databases such as TIMER 2.0, UALCAN, LinkedOmics and in vitro experiments.

**Results:**

It was found that the expression of TCIRG1, was significantly increased in ccRCC tissue, and that high TCIRG1 expression was associated with poor overall survival (OS) and short progression-free interval (PFI). In addition, TCIRG1 expression was highly correlated with the infiltration immune cells, especially CD4^+^T cell Th1, CD8^+^T cell, NK cell, and M1 macrophage, and positively correlated with PDCD1, CTLA4 and other immunoinhibitors, CCL5, CXCR3 and other chemokines and chemokine receptors. More importantly, TCIRG1 may regulate aerobic glycolysis in ccRCC via the AKT/mTOR signaling pathway, thereby affecting the malignant progression of ccRCC cell lines.

**Conclusions:**

Our results demonstrate that the glycolysis-related biomarker TCIRG1 is a tumor-promoting factor by affecting aerobic glycolysis and tumor immune microenvironment in ccRCC, and this finding may provide a new idea for the treatment of ccRCC by combination of metabolic intervention and immunotherapy.

**Supplementary Information:**

The online version contains supplementary material available at 10.1186/s12935-023-03019-0.

## Background

Renal cell carcinoma (RCC) is one of the most common malignancies of the genitourinary system [1] and the most common solid lesion in the kidney, accounting for approximately 90% of all renal malignancies [2]. The principle of clinical treatment for localized tumors is nephrectomy or radical nephrectomy followed by postoperative individualized and precise adjuvant therapy to reduce the risk of tumor recurrence and metastasis and improve the postoperative survival rate. Based on current evidence, smoking tobacco, obesity and hypertension remain established risk factors for renal cancer [3, 4]. Renal cancer is considered to be one of the models for studying metabolic reprogramming [1, 5–8]. During renal tumorigenesis, genes which are mutated, inactivated, or hyperactivated involved in regulating metabolic events such as glycolysis, tricarboxylic acid (TCA) cycle, glutamine metabolism, and lipid metabolism [1, 5–8]. As a result, renal cancer has been referred to as a “Metabolic Disease” [8, 9]. In ccRCC, the enzymes responsible for replenishing the metabolic flux to the TCA cycle from other pathways are frequently down-regulated [10]. These pathways include glycolysis, lipid metabolism, and glutamine metabolism [10]. Research has indicated that advanced stages of ccRCC are linked to elevated levels of glutamine and increased activity in the glutathione/oxidized glutathione pathways [10]. Additionally, ccRCC patients often exhibit higher levels of cholesterol ester accumulation in their kidneys [10]. In recent years, metabolic signatures associated with RCC has also stimulated interest in targeted metabolism as a novel therapeutic strategy and in the treatment of RCC, the first metabolic target is mammalian target of rapamycin (mTOR), in addition to promoting HIF1 translation, mTOR complex 1 also drives protein and lipid processing by intercepting signals from glucose, growth factors, and amino acids [5]. Abnormal upregulation of glycolysis signals in RCC can promote tumor growth and tumor cell interaction with immune cells in the immune microenvironment (IME), resulting in an imbalance between pro-tumor and anti-tumor immunities, resulting in suppression of the IME, which mediates tumor immune escape [11]. ​RCC consists of three major histological subtypes, in particular ccRCC is most closely related to glycolysis [8]. In previous studies, RCC of different tissue subtypes have been compared by using gene expression signatures of the major metabolic pathways [8]. Expression levels of the gene signatures for the Krebs cycle and the electron transport chain (ETC) were low in ccRCC compared with intermediate expression in papillary RCC (pRCC) and high expression in chromophobe RCC (chRCC). In ccRCC, the loss of gene expression of the Krebs cycle and ETC was paired with an increased expression of glycolysis pathway genes that is consistent with the warburg effect of aerobic glycolysis and suppression of oxidative phosphorylation [8]. In an era of rapid advances in advanced renal cancer treatment, immune checkpoint inhibitors are gaining ground to replace anti-VEGFR-TKI as a current first-line treatment [12]. Therefore, further understanding of the role of tumor glucose metabolism in the IME is of great significance to explore biomarkers of tumor immune infiltration in glycolysis and improve the efficacy of immunotherapy.

Tumor initiation and progression are closely related to tumor metabolism and the tumor microenvironment (TME) [13]. Tumor cells reprogram their metabolism to promote tumor growth, metastasis and survival. They exhibit a dependence on glycolysis, mainly manifested as increased glucose uptake and lactate to meet the increased anabolic demands of cancer cell proliferation [14]. This metabolic reprogramming provides sufficient energy for tumor cells, promotes their growth and proliferation, and helps tumor cells escape [15]. Tumor cells and tumor-infiltrating T lymphocytes compete for glucose, and massive glucose consumption by tumor cells change the metabolic microenvironment of T lymphocytes, inhibit IFN-γ, and promote tumor progression and immune escape [16]. Changes in cancer cell metabolism provide insights into the development of specific therapeutic targets and anticancer drugs. Currently, therapeutic strategies for glycolysis and cancer cell-specific biosynthetic pathways have become a major focus of cancer research. The increased dependence of tumor cell glycolysis suggests a potential therapeutic effect of glycolytic inhibitors in cancer therapy, but glycolytic inhibition alone is ineffective in clinical practice [17]. Therefore, regulating metabolism in combination with immunotherapy is expected to improve treatment response and may help overcome drug resistance [18]. The enhancement of aerobic glycolysis in tumor cells and its by-product, lactic acid, can regulate tumor matrix and tumor immune microenvironment, and lactic acid can induce the polarization of tumor-associated macrophages (TAM) into M2-like type, thus promoting tumor progression [19]. Targeting glycolytic changes in the TME has been shown as a safe and effective strategy to improve therapeutic efficacy [20, 21]. T-cell immune regulator 1 (TCIRG1), also known as V-type proton atpase 116 kDa subunit a3 or T-cell immune response cDNA 7 protein (TIRC7), was first identified in osteosclerosis, and mutations in TCIRG1 are a common cause of human autosomal recessive osteosclerosis [22]. Previous studies reported that up-regulation of TIRC7 could prevent human T cell proliferation and interleukin-2 (IL-2) secretion, and anti-TIRC7 antibody could specifically inhibit membrane protein encoding, thus enabling crucial type 1 subtype-specific IFN-γ expression [23]. In addition, in hepatocellular carcinoma (HCC), TCIRG1 can act as a metastasis enhancer by regulating HCC cell growth, death and epithelial–mesenchymal transition (EMT), and may also be a therapeutic target for cancer and metastasis [24].

The aim of the present study was to screen the glycolysis-related biomarker TCIRG1 associated with immune infiltration by bioinformatics analysis based on The Cancer Genome Atlas (TCGA) database and MSigDB database. Our preliminary validation of HPA, TIMER 2.0, UALCAN and other databases suggested that high TCIRG1 expression was associated with poor prognosis in ccRCC patients and verified the correlation between TCIRG1 expression and immune features in ccRCC. Furthermore, we explored the effects of the glycolysis-related biomarker TCIRG1 on the proliferation, migration, invasion and apoptosis of ccRCC in vitro. The effect of TCIRG1 on glycolytic metabolism in ccRCC and its relation to the AKT/mTOR signaling pathway were also investigated. Our study may provide a tumor immunobiomarker that could affect aerobic glycolysis in ccRCC.

## Materials and methods

### Public datasets acquisition

Gene expression data and corresponding clinical information were obtained from The Cancer Genome Atlas (TCGA) public database provided by UCSC Xena (https://xenabrowser.net/, accessed on August 2, 2022) [25]. A total of 607 samples were analyzed, including 535 ccRCC samples and 72 normal kidney tissue or adjacent tissue samples. We excluded patients who lacked OS time or PFI time, after removing patients who were not eligible, 525 patients were finally left for analysis. They were randomized to a training cohort and a validation cohort in an approximate 1:1 ratio. Their clinical characteristics are shown in Table 1. Proteomic expression data and corresponding clinical information were obtained from 232 tumor and adjacent non-tumor tissue pairs from Chinese ccRCC patients [26], and their expression data and clinical information were shown in Supplementary Table 3. The primary outcomes were OS and PFI. OS was defined as the follow-up time from surgery to the date of death or the last clinical visit. PFI was defined as survival without further disease progression after treatment, and the outcome measure was tumor death. We include the ccRCC of scRNA-seq for the analysis of TCIRG1 scRNA expression in renal tumor and normal renal tissues, and their expression data and clinical information were shown in Supplementary Tables 4 [27]. All the final raw count matrices were analyzed by R software version 4.1.3, and p < 0.05 was considered statistically significant.

**Table 1:** Clinicopathologic characteristics of patients with clear cell renal cell carcinoma (ccRCC) (n = 525)

Characteristics	Training cohort	Validation cohort	Combined cohort
	(n = 262)	(n = 263)	(n = 525)
Age			
<60	119	125	244
≥ 60	143	138	281
Gender			
Male	180	163	343
Female	82	100	182
TNM stage			
I-II	158	159	317
III-IV	102	103	205
NA	2	1	3
Pathological grade			
1–2	117	121	238
3–4	140	139	279
NA	5	3	8
Overall survival			
Alive	181	173	354
Dead	81	90	171
Progression Free Interval			
Free of progression	186	179	365
Progressed	76	84	160

### Gene set enrichment analysis (GSEA)

**MSigDB** (http://www.gsea-msigdb.org/gsea/index.jsp) is a resource of tens of thousands of annotated gene sets for use with GSEA software, divided into Human and Mouse collections [28]. Combined with the selection strategy of previous studies related to glycolysis [29], we used “glycolysis” as the search term in the MSigDB database and searched 21 glycolysis-related gene sets. These gene sets included hallmark glycolysis, reactive body glycolysis, and others. The details are described in Supplementary Table 2. In addition, the extracted glycolysis-related genes were intersected with KIRC up-regulated differentially expressed genes (DEGs) to obtain glycolysis-related differentially expressed genes (DEGs) in KIRC. We also used GSEA 4.2.3 software to investigate potential pathways for the activation of 6 DEGs associated with glycolysis in KIRC. When both normalized enrichment score (NES) > 1 and false discovery rate (FDR) q value < 0.05 were satisfied, the number of permutations was set to 1000 and the gene set was considered significantly enriched in RCC samples.

### Protein-protein interaction (PPI) network, Cox regression analysis and ROC curve

**The String** (https://cn.string-db.org/) database was used to further analyze the interactions between 124 glycolysis-related genes [30]. Cytoscape software was used for network visualization to screen 124 hub genes associated with glycolysis. Knowing that relevant factors such as patients, tumors and treatment are associated with OS, we further performed univariate and multivariate analyses of the remaining glycolysis-related hub genes using Cox regression models, and then used the “Forest Map” package to display the p value, hazard ratio (HR), and 95% confidence interval (CI) for each variable in the forest map. P < 0.05 was considered statistically significant. Finally, the diagnostic value of the 19 glycolysis-related hub gene expressions was evaluated by using receiver operating characteristic (ROC) curve. Knowing that a high area under curve (AUC) corresponds to a high predictive power, we selected hub genes with AUC value > 0.9 for further analysis.

### Public database analysis

Public databases including HPA, TIMER, TIMER 2.0, UALCAN, LinkedOmics, TISIDB and TISCH2 were used to analyze and visualize the expression of TCIRG1 and its relationship with immune cell infiltration level based on KIRC data set (n = 533).

**HPA** (https://www.proteinatlas.org/) Among them, tissue and pathological maps provide information about the expression profiles of specific genes in normal and tumor tissues at the protein level. All tissue images in HPA database were stained by immunohistochemistry [31].

**TIMER** (https://cistrome.shinyapps.io/timer/) Correlations between gene expression (log2TPM) in the KIRC dataset and infiltration of 6 immune cells (infiltration estimates) were shown based on Gene module. P < 0.05 was considered statistically significant [32].

**TIMER 2.0** (http://timer.cistrome.org/) Correlation between TCIRG1 gene expression (log2TPM) and immune cell infiltration (infiltration estimates) in KIRC dataset was analyzed by XCELL algorithm based on Gene module. Based on the Gene_Corr module analysis, the correlation between the expression of TCIRG1 and the indicated immune cell marker genes was shown. The partial Spearman’s correlation was used to perform this association analysis. P < 0.05 was considered statistically significant [33].

**UALCAN** (http://ualcan.path.uab.edu/) Based on KIRC or ccRCC data sets, we analyzed the differences of TCIRG1 mRNA in tumor differentiation level, lymph node metastasis stage, different tumor stages, and the differences of TCIRG1 proteomic expression. The degree of tumor differentiation was defined as: Grade 1, well differentiated; Grade 2, moderately differentiated; Grade 3, poorly differentiated; Grade 4, undifferentiated [34].

**CellMarker** (http://xteam.xbio.top/CellMarker/) It provides a user-friendly interface for browsing, searching and downloading markers of diverse cell types of different tissues [35].

**LinkedOmics** (http://www.linkedomics.org/login.php) This database was used to perform and visualize biological processes in GO analysis and KEGG analysis to demonstrate TCIRG1 enrichment in biological processes and pathways [36].

**TISIDB** (http://cis.hku.hk/TISIDB/) Correlations between KIRC gene expression and immunoinhibitors, chemokines, and chemokine receptors were analyzed [37].

**TISCH2** (http://tisch.comp-genomics.org/home/) We used this database to perform single cell analysis to investigate the TCIRG1 expression in different immune cell types [38].

We also used the ESTIMATE algorithm to assess the Stromal Score, Tumor Purity, Immune Score and Estimate Score of TCIRG1.

### Cell culture

The RCC cell lines used in this study were obtained from the cell bank of the Typical Culture Preservation Center of the Chinese Academy of Sciences (Shanghai, China) in 2020. All cells were cultured according to the recommended procedures according to the American Type Culture Collection (ATCC) as we reported in a previous study [39]. In short, HK-2(ATCC, CRL-2190) cells were cultured in Dulbecco’s modified Eagle’s medium (DMEM), a high sugar medium (Gibco). ACHN (ATCC, CRL-1611) and A498 (ATCC, HTB-44) cells were cultured in Minimum Essential Medium (Gibco). 786-O (ATCC, CRL-1932), 769-P (ATCC, CRL-1933) and OS-RC-2 cells were kept in RPMI-1640 medium (Gibco). All culture media were supplemented with fetal bovine serum (FBS, 10%, Gibco) and 1% penicillin/streptomycin (Gibco). All cell lines were cultured at 37 °C and 5% CO_2_. All cell lines used in this study were cultured within 40 passages.

### Gene knockdown

The general procedure of this section was previously reported [40]. Briefly, the OS-RC-2 cells or 769-P cells were cultured in 6-well plates, inoculated at a density of 5 × 10^4^ cells/ml, and transfected with the small interference RNA (siRNA) of TCIRG1 or negative control siRNA (negative control, NC) using Lipofectamine 3000 reagents (L3000015, Invitrogen) according to the manufacturer’s introductions. After 72-h transfection, they were harvested for further experiments after RT-qPCR validation of transfection efficacy and specificity (supplementary Figure S2K). The sequences for siRNA are as follow:

TCIRG1 siRNA 1:

F: GGGUGGAAUUCCAGAACAAGU,

R: UUGUUCUGGAAUUCCACCCAG;

TCIRG1 siRNA 2:

F: GCGUGAGCACCACGCACAAGU,

R: UUGUGCGUGGUGCUCACGCUG;

TCIRG1 siRNA 3:

F: AGAUGAAGAUGUCCGUCAUCC,

R: AUGACGGACAUCUUCAUCUUG.

### Real time flurocent qualitative PCR (RT-qPCR)

RT-qPCR assay was performed according to the manufacturer’s instructions as we previously described [41]. Total RNA was extracted with Trizol reagents (Invitrogen) and cDNA was obtained using First-Strand cDNA Synthesis Kit (Invitrogen). The resulting cDNA was subjected to RT-qPCR with the indicated primer sets. RT-qPCR analysis was conducted by Power SYBR Green PCR Master Mix (Applied Biosystems, Foster City, CA, USA). Relative gene expression was normalized to GAPDH with the ^2−^ΔΔCT assay. The primer sequences used are as follows: TCIRG1-F: CCGTGATGACCGTGGCTATCCT; TCIRG1-R: CATCTGTGGCAGCGAAGGTGAA; GAPDH-F: TCAGACACCATGGGGAAGGT; GAPDH-R: CTTCCCGTTCTCAGCCATGTA.

### Western blot

As we reported earlier [41], western blot analysis was performed, total protein was firstly extracted using SDS-PAGE and then transferred to a PVDF membrane (Termo, USA). Afterwards, the PVDF membrane was incubated with antibody: TCIRG1 (12649-1-AP, proteintech), GAPDH (#5174S, cell signal technology), AKT3 + AKT2 + AKT1 (ab32505, abcam), p-AKT (28731-1-AP, proteintech), mTOR (28273-1-AP, proteintech), and p-mTOR (80596-1-RR, proteintech).

### Cell proliferation assays

The proliferation of RCC cells was measured using the CCK-8 kit (Dojindo) according to the manufacturer’s instructions. 1 × 10^3^ cells were cultured in each well of the 96-well plate. After adherence, 10 ul CCK-8 was added to each well, and the cell samples were then incubated at 37 °C for 2 h. The optical density (OD) value was recorded at 450 nm with a microplate reader (EXL800, BioTek Instruments). The proliferation rates are expressed as a proportion of the control value, which was obtained from the normal control (NC) groups.

### Transwell assays

800 μl 1640 (including 10% FBS) was put in the bottom wells, and about 1.5 × 10^4^ cells resuspended in 200 μl serum-free medium were put into the upper chambers. In addition, the invasion assay was carried out with 100 μl 1:8 diluted matrixgel in the upper chambers. After 36 h incubation with 5% CO_2_ at 37 ℃, cells were treated with 4% paraformaldehyde at room temperature for 20 min, stained with 0.1% crystal violet at room temperature for 30 min, washed with PBS, counted and photographed under the light microscope.

### Apoptosis assessment

Apoptotic cells were evaluated through ANNXIN-V FITC and PI staining (Beyotime, C1062L) according to the manufacturer’s instructions, and then analyzed by flow cytometry (FACS Calibur).

### Metabolism assays

Cells were seeded in a 6-well plate and cultured for 24 h. The medium was collected and tested for glucose, lactate and pyruvate with the glucose assay kit, lactate assay kit and pyruvate assay kit, respectively. Intracellular ATP was detected using the ATP determination kit (Nanjing Jian Cheng Bioengineering Institute, Nanjing, China) according to the manufacturer’s protocol. The results were normalized by protein concentration, and triple-independent experiments were performed.

### Statistical analysis

Statistical analysis and graphic visualization of data were performed with R 4.1.3, GraphPad Prism 8.0 and SPSS 22.0 (IBM corporation) software. Numerical data are expressed as the mean ± standard deviation (SD). Paired Student’s t-test was used to analyze mRNA levels of TCIRG1 in RCC cell lines. Pearson or Spearman coefficients were used to calculate correlations between variables. Independent Student’s t-tests were used to compare all statistical calculations, including the cell growth rate, glucose consumption, lactate production, pyruvate production, ATP production and TCIRG1 scRNA expression. Results with p < 0.05 were considered statistically significant.

## Results

### 7 Glycolysis-related hub genes are identified as independent prognostic indicators in KIRC

**Fig. 1:**
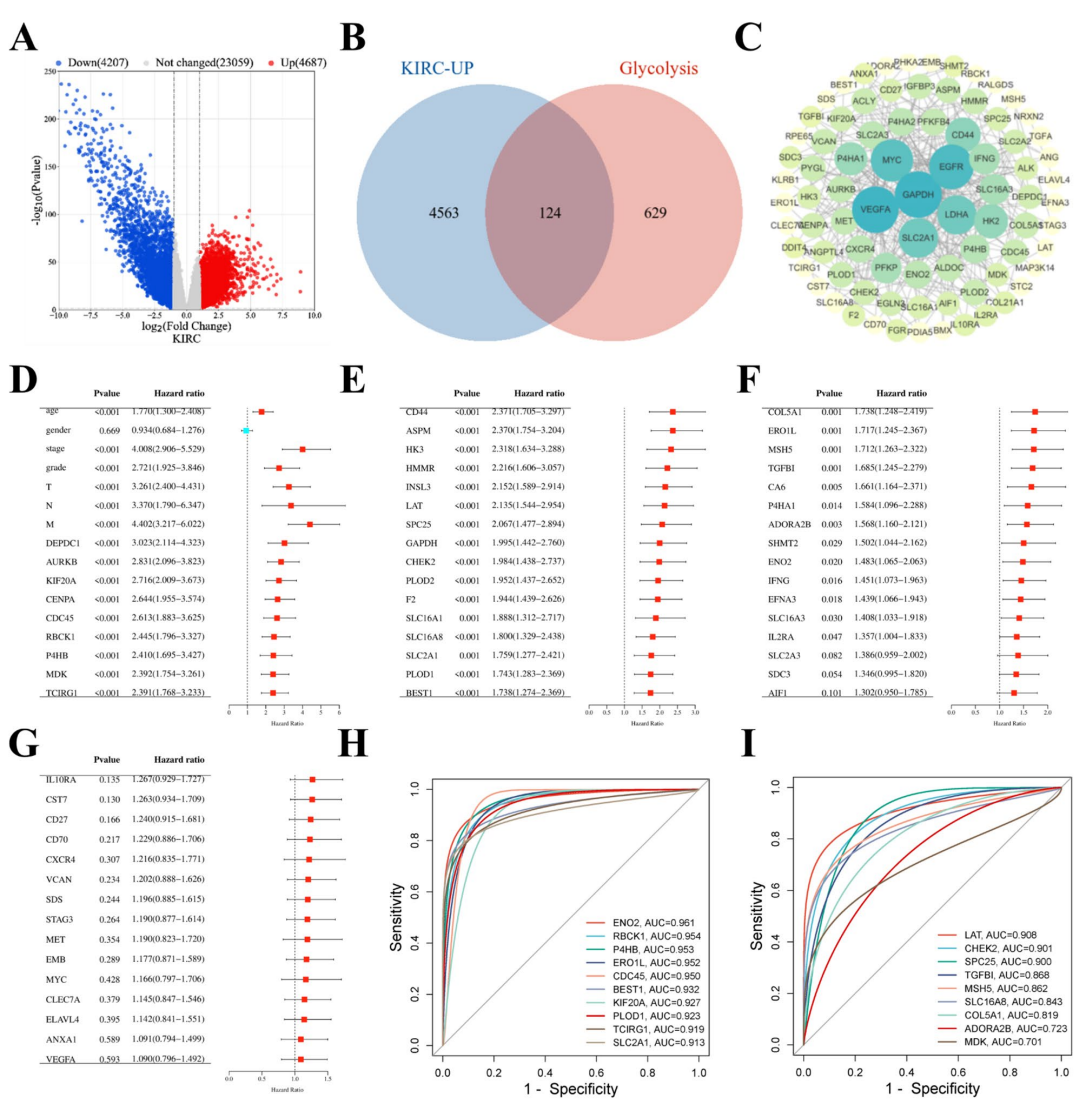
Glycolysis-related hub genes were screened by TCGA database and MSigDB database. (**A**) In TCGA-KIRC (|log (FC)|>1, P < 0.05), compared with normal tissues, 4687 up-regulated genes and 4207 down-regulated genes were selected. (**B**) The intersection of 4687 up-regulated genes and 753 glycolysis-related genes was used to select 124 glycolysis-related differentially expressed genes (DEGs). (**C**) By analyzing the association between 124 glycolysis-related DEGs, a PPI network was established, and 86 hub genes were ultimately screened. (**D**-**G**) Incorporating 86 hub genes and 7 clinicopathological information into univariate cox regression analysis, 38 hub genes were selected. (**H**-**I**) Incorporating 19 hub genes into the receiver operating characteristic (ROC) curve, and selecting 12 hub genes with area under curve (AUC) > 0.9. TCGA, The Cancer Genome Atlas; MSigDB, Molecular Signatures Database; KIRC, Kidney Renal clear Cell Carcinoma; DEGs, differentially expressed genes; PPI, Protein-Protein interaction; ROC, receiver operating characteristic; AUC, Area under curve

By analyzing the gene expression profiles and corresponding clinical data of 535 RCC samples and 72 normal kidney tissue controls from TCGA, we obtained 8894 DEGs by using R, including 4687 up-regulated genes and 4207 down-regulated genes (Fig. 1A). Then, 21 glycolysis-related gene sets were searched from the MSigDB database, including 753 glycolysis-related genes (Supplemental Table 2). In order to search for biomarkers that promoted tumor progression, we intersected 4687 up-regulated DEGs with 753 glycolysis-related genes and screened out 124 glycolysis-related DEGs (Fig. 1B). To clarify the associations between these glycolysis-related genes, we constructed a PPI network for 124 glycolysis-related DEGs using the String database and Cytoscape software. We then screened 86 glycolysis-related hub genes in RCC (Fig. 1C). To further screen for glycolysis-related hub genes affecting the progression of RCC, we included 86 glycolysis-related hub genes and 7 clinical parameters in the univariate cox regression analysis. The results showed that age, stage, grade, TNM stage, and the high expression of 38 glycolysis-related hub genes predicted poor OS in KIRC (Fig. 1D-G). Therefore, we further included 38 glycolysis-related hub genes and 6 clinical parameters with p < 0.05 into the multivariate cox regression analysis. The results showed that 19 glycolysis-related hub genes were independent prognostic indicators of poor OS in KIRC (Supplementary Figure S1A). The diagnostic potential of the 19 glycolysis-related hub genes for KIRC was estimated by ROC curve (Fig. 1H-I), and 12 genes with AUC > 0.9 were selected for further analysis. To screen out glycolysis-related hub genes with histological differences between normal renal tissue and renal tumor tissue, we analyzed 12 glycolysis-related hub genes previously screened through HPA database, and the results showed that the protein expression level of ENO2, P4HB, CDC45, TCIRG1, SLCA1, ERO1A and PLOD1 in renal tumor tissue was higher than that in normal renal tissue (Supplementary Figure S1B). However, the protein expression level of BEST1, KIF20A, CHEK2 and LAT in normal renal tissue was higher than in renal tumor tissue (Supplementary Figure S1C), while there were no data for RBCK1 to be analyzed. So far, the 7 glycolysis-related hub genes were highly expressed in renal tumor tissue and were identified as independent prognostic indicators in renal tumor tissues.

### The glycolysis-related hub gene TCIRG1 is associated with the immune response in ccRCC

**Fig. 2:**
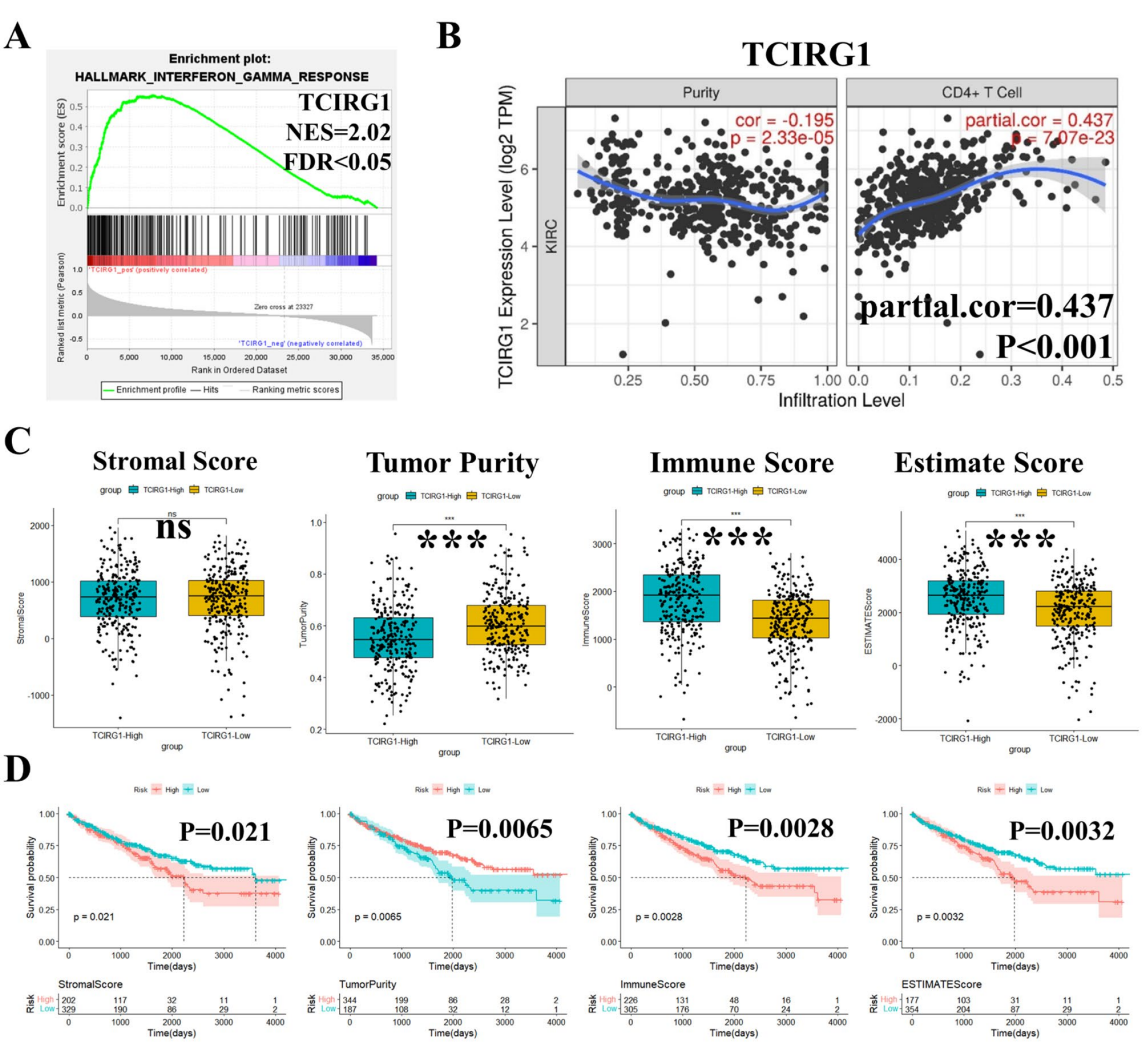
Correlation between glycolysis-related hub gene TCIRG1 and immune response. (**A**) GSEA enrichment analysis shows that TCIRG1 is significantly enriched on the Interferon gamma response. (**B**) TIMER database shows the correlation between TCIRG1 expression level (log2 TPM) and CD4 + T Cell (Infiltration Level). (**C**) Correlation between the expression of TCIRG1 and the immune score of ccRCC (Stromal Score, Tumor Purity, Immuno Score, and Estimate Score) based on Pearson correlation analysis. (**D**) K-M survival curve, relationship between OS of ccRCC and Stromal Score, Tumor Purity, Immune Score, and Estimate Score. (all p values were defined as *p < 0.05, **p < 0.01 and ***p < 0.001, log rank test). TCIRG1, T-cell immune regulator 1; GSEA, Gene Set Enrichment Analysis; TIMER, Tumor Immune Estimation Resource; K-M, Kaplan-Meier; ccRCC, clear cell renal cell carcinoma; OS, Overall survival

To explore glycolysis-related biomarkers associated with immunotherapy response, we included 7 previously selected glycolysis-related DEGs into the GSEA software for enrichment analysis, and found that 6 DEGs were mainly associated with interferon gamma response (Fig. 2A, Supplementary Figure S2A). Knowing that activation of T cells, especially by CD4^+^T (Th1) cells, can activate IFN-γ [42], we further screened TCIRG1 through the online TIMER database and found that it had the highest correlation with CD4^+^T cell immune infiltration (partial.cor = 0.437, P < 0.001) (Fig. 2B, Supplementary Figure S2B). In tumors, the ratio of immune cells to stromal cells also has a significant effect on prognosis, which is vital for tumor diagnosis and prognostic assessment [43]. Therefore, we used the ESTIMATE algorithm to calculate Stromal Score, Tumor Purity, Immune Score, and Estimate Score in ccRCC tissue based on the TCGA transcriptome data, and found that they had a significant correlation with TCIRG1 expression (Fig. 2C). In addition, higher Immune and Estimate Scores and lower Tumor Purity were associated with poorer OS in ccRCC patients (Fig. 2D), suggesting that the expression of TCIRG1 may be associated with changes in the TME during the diagnosis and prognosis of ccRCC.

### High expression of TCIRG1 in KIRC is associated with poor prognosis

**Fig. 3:**
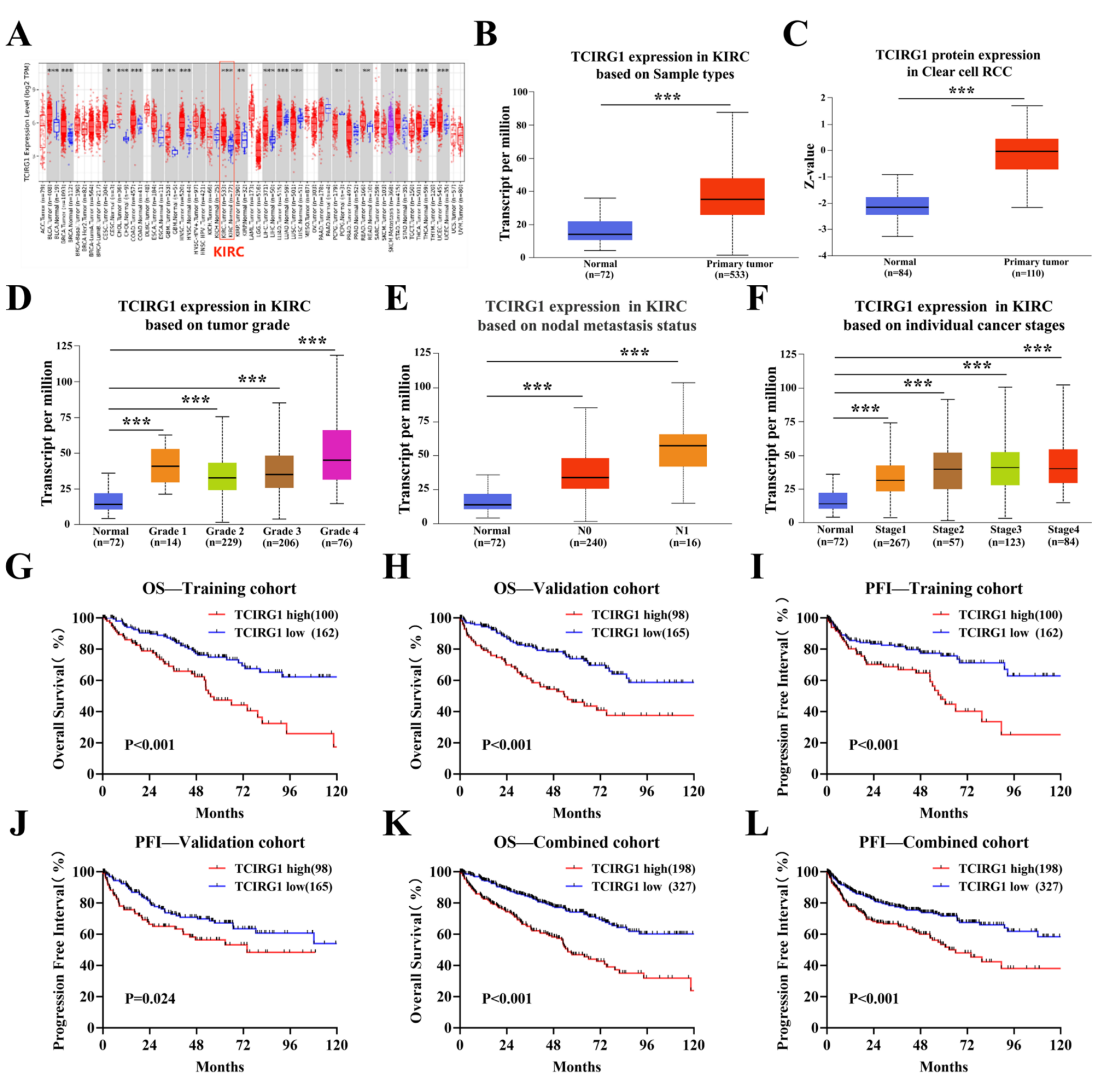
Differential expression of TCIRG1 in renal tumor tissue and normal renal tissue and Kaplan-Meier survival analysis of Overall survival(OS)and Progression Free Interval (PFI) in KIRC patients. (**A**) The TIMER 2.0 Gene_DE module was used to detected the difference in TCIRG1 mRNA expression between tumor tissues and adjacent normal tissues in TCGA cancer types. The distribution of gene expression levels is shown in a box plot. The result of the KIRC dataset is marked with a red box. (**B**-**C**) UALCAN analysis showed that in the KIRC dataset of TCGA, the differential expression of TCIRG1 mRNA in primary tumor tissue and adjacent normal tissue was analyzed; In the ccRCC dataset of CPTAC, there is a difference in the expression of TCIRG1 protein between primary tumor tissue and adjacent normal tissue. (**D**-**F**) UALCAN analysis showed that in the KIRC dataset, there were differences in the expression of TCIRG1 at different tumor differentiation levels (**D**), lymph node metastasis (**E**), and different tumor stages (**F**). Tumor differentiation levels are defined as: Grade 1, well-differentiated; Grade 2, moderately differentiated; Grade 3, poorly differentiated; Grade 4, undifferentiated. There are differences in the expression of undifferentiated tumor differentiation level (**E**) and lymph node metastasis (**F**). (**G**-**L**) K-M survival curve analysis showed that in the training cohort (n = 262; **G**, **I**), validation cohort (n = 263; **H**, **J**), and combined cohort (n = 525; **K**, **L**), KIRC patients with high TCIRG1 expression had short OS and PFI. (all p values were defined as *p < 0.05, **p < 0.01 and ***p < 0.001, log rank test). KIRC, Kidney Renal Carcinoma; mRNA, messenger RNA; UALCAN, The University of ALabama at Birmingham Cancer data analysis Portal; CPTAC, Clinical Proteomic Tumor Analysis Consortium

Next, we explored the expression of TCIRG1 in different normal and tumor tissues using the TIMER 2.0 database, and found the expression of TCIRG1 in renal tumor tissues was higher than that in normal renal tissues (Fig. 3A). This finding is consistent with the KIRC data set based on TCGA and CPTAC samples (Fig. 3B-C). In addition, we also investigated the relationship between TCIRG1 expression and the clinicopathological characteristics in KIRC patients, and found that the expression level of TCIRG1 was higher in KIRC patients with poorer tumor differentiation, lymph node metastasis, and high clinical stage (Fig. 3D-F). To further investigate the effect of TCIRG1 expression on OS and PFI in KIRC patients, they were randomly divided into a training cohort and a validation cohort in a ratio of approximately 1:1 (Table 1). Patients in the three cohorts were then divided into a TCIRG1 high-expression group and a TCIRG1 low-expression group according to the optimal cut-off value of TCIRG1 (Supplementary Tables S1-S3). Similar to the previous analysis results, in the training cohort (Fig. 3G, I), validation cohort (Fig. 3H, J), and combined cohort (Fig. 3K, L), patients with high TCIRG1 expression had shorter OS and PFI than those with lower TCIRG1 expression, indicating that high TCIRG1 expression was associated with poor prognosis in KIRC patients, and demonstrated that TCIRG1 expression could be an independent risk factor (Tables 2 and 3, Supplementary Tables S4). And we analyzed a proteogenomic data of clear cell renal cell carcinoma in a Chinese population, it was also found that high TCIRG1 expression predicted poor OS in ccRCC patients (Supplementary Figure S2C) [26].

**Table 2:** Univariate and multivariate Cox regression analysis of TCIRG1 expression classifier and clinical characteristics with Overall Survival and Progression Free Interval in training cohort (n = 262)

Characteristics	Overall survival						Progression Free Interval					
	Univariate			Multivariate			Univariate			Multivariate		
	HR (95%CI)	p Value		HR (95%CI)	p Value		HR (95%CI)	p Value		HR(95%CI)		p Value
Age (≥ 60 vs. < 60)	1.836(1.155–2.918)	0.010		1.626(1.018–2.596)	0.042		1.317(0.831–2.087)	0.242				
Gender (Male vs. Female)	1.121(0.695–1.808)	0.639					1.891(1.088–3.288)	0.024		1.944(1.106–3.417)		0.021
TNM stage (III-IV vs. I-II)	3.262(2.060–5.166)	< 0.001		2.248(1.365–3.701)	0.001		6.314(3.738–10.664)	< 0.001		5.197(2.977–9.072)		< 0.001
Pathological grade (III-IV vs. I-II)	2.800(1.673–4.687)	< 0.001		1.766(1.016–3.701)	0.044		4.079(2.276–7.308)	< 0.001		2.340(1.284–4.267)		0.006
TCIRG1 expression (High vs. Low)	2.409(1.554–3.736)	< 0.001		1.679(1.061–2.658)	0.027		2.085(1.319–3.297)	0.002		1.315(0.813–2.126)		0.265

* TCIRG1 was divided into high and low expression groups with an optimal cutoff value of 3.82

TCIRG1 low expression: Gene expression level of TCIRG1 < 3.82;

TCIRG1 high expression: Gene expression level of TCIRG1 ≥ 3.82;

Statistical significance was calculated by Chi squared test or Fisher’s exact test for categorical/binary measures

Abbreviation: ccRCC, clear cell renal cell carcinoma

**Table 3:** Univariate and multivariate Cox regression analysis of TCIRG1 expression classifier and clinical characteristics with Overall Survival and Progression Free Survival in validation cohort (n = 263)

Characteristics	Overall survival							Progression Free Interval							
	Univariate				Multivariate			Univariate				Multivariate			
	HR (95%CI)	p Value			HR (95%CI)	p Value		HR (95%CI)	p Value			HR(95%CI)		p Value	
Age (≥ 60 vs. < 60)	1.729 (1.123–2.663)		0.013		1.398(0.898–2.178)	0.138		1.379(0.892–2.132)	0.149						
Gender (Male vs. Female)	0.817(0.537–1.242)		0.344					1.265(0.798–2.006)	0.317						
TNM stage (III-IV vs. I-II)	4.760(3.028–7.481)		< 0.001		3.509(2.159–5.701)	< 0.001		7.036(4.274–11.581)	< 0.001			5.868(3.476–9.907)		< 0.001	
Pathological grade (III-IV vs. I-II)	2.467(1.554–3.915)		< 0.001		1.432(0.878–2.335)	0.150		3.061(1.864–5.026)	< 0.001			1.994(1.171–3.394)		0.011	
TCIRG1 expression (High vs. Low)	2.373(1.566–3.596)		< 0.001		1.679(1.086–2.596)	0.020		1.658(1.075–2.559)	0.022			0.897(0.568–1.419)		0.643	

* TCIRG1 was divided into high and low expression groups with an optimal cutoff value of 3.82

TCIRG1 low expression: Gene expression level of TCIRG1 < 3.82;

TCIRG1 high expression: Gene expression level of TCIRG1 ≥ 3.82;

Statistical significance was calculated by Chi squared test or Fisher’s exact test for categorical/binary measures

Abbreviation: ccRCC, clear cell renal cell carcinoma

### Correlation between TCIRG1 expression and immune characteristics in ccRCC

**Fig. 4:**
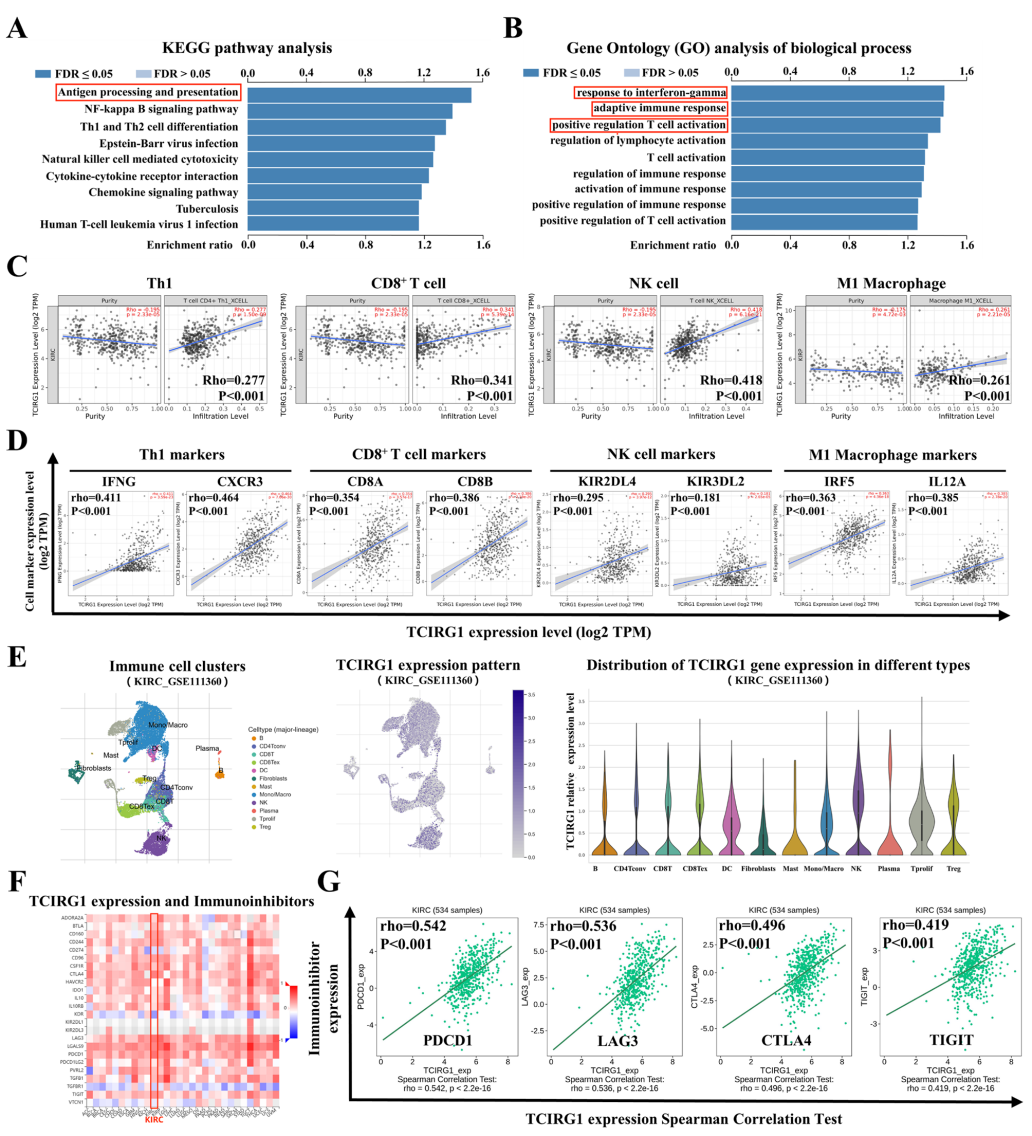
Correlation between expression of TCIRG1 and immune characteristics of ccRCC. (**A**) LinkedOmics database analysis shows that in the KEGG pathway, TCIRG1 was enriched in immune related pathway antigen processing and presentation. (**B**) Biological processes in gene ontology (GO) analysis indicates that TCIRG1 is enriched in immune related biological processes, including response to interferon-gamma, adaptive immune response, positive regulation T cell activation. (**C**) TIMER 2.0 Gene_DE module analysis shows the correlation between TCIRG1 gene expression (log2 TPM) and indicated immune cell infiltration level in the KIRC dataset based on the XCELL algorithm. The partial Spearman’s correlation is used to perform this association analysis. (**D**) TIMER 2.0 Gene_Corr module analysis shows that TCIRG1 expression was correlated with the indicated immune cell marker genes (Th1: IFNG, CXR3; CD8 + T cell: CD8A, CD8B; NK cell: KIR2DL4, KIR3DL2; M1 macrophage: IRF5, IL12A). (**E**) Single-cell expression matrix and corresponding statistical chart from TISCH2 database illustrating the expressive abundance of TCIRG1 in different clusters of immune cells based on KIRC_GSE111360 data set. (**F**-**G**) TISIDB analysis shows a correlation between TCIRG1 expression and Immunoinhibitors in the KIRC dataset. KEGG, Kyoto Encyclopedia of Genes and Genomes; TISCH2, Tumor Immune Single-cell Hub 2; TISIDB, Tumor-immune system interactions data types

The above results demonstrated that the glycolysis-related biomarker TCIRG1 was associated with the immune therapy response through a series of approaches. To further explore the relationship between TCIRG1 and the immune properties of ccRCC, we analyzed the LinkedOmics database and found that the expression of TCIRG1 was closely related to the immune process via the KEGG pathway and the biological process of GO in RCC. In addition, TCIRG1 was associated with antigen processing and presentation, response to interferon-gamma, adaptive immune response, and positive regulation T cell activation (Fig. 4A, B). Then, we used the XCELL algorithm of TIMER 2.0 to evaluate the relationship between TCIRG1 expression and infiltration of different immune cell types in ccRCC. Scatter plots showed that TCIRG1 expression was significantly positively correlated with the infiltration of CD4^+^T cell Th1 (Rho = 0.277, P < 0.001), CD8^+^T cell (Rho = 0.341, P < 0.001), NK cell (Rho = 0.418, P < 0.001), and M1 macrophage (r = 0.261, P < 0.001) (Fig. 4C), suggesting that TCIRG1 may promote the tumor immune response in ccRCC by positively regulating CD4^+^T cell Th1, CD8^+^T cell, NK cell, and M1 macrophage. To explore the correlation between TCIRG1 expression and immune cell markers, we determined the markers of immune cells based on the CellMarker database, and then evaluated TCIRG1 expression and Th1 markers (IFNG and CXCR3), CD8^+^T cell markers (CD8A and CD8B), NK cell markers (KIR2DL4 and KLR3DL2), and M1 macrophage markers (IRF5 and IL12A) through the TIMER 2.0 database (Fig. 4D). The results suggest that TCIRG1 was significantly correlated with markers of four immune cells, especially with markers of CD4^+^T cell Th1. We also found that TCIRG1 was mainly localized in mononuclear macrophage and NK cell based on the single cell RNA-seq (scRNA-seq) dataset GSE11136 of the TISCH2 database, which is consistent with our previous results (Fig. 4E). In addition, we used the scRNA-seq dataset GSE139555 from renal cancer patients and found that TCIRG1 expression was higher in renal tumors tissues than in normal renal tissues (Supplement Figure S2D-F) [27], which is consistent with our results in TCGA-KIRC. Knowing that immunocheckpoint inhibitors (ICIs) are a significant new group of tumor immunotherapy drugs [44], we used the TISIDB database to analyze the correlation between the expression level of TCIRG1 and ICIs in different human cancer types (Fig. 4F). The heat map results showed that TCIRG1 was significantly positively correlated with the expression of some ICIs in KIRC including PDCD1 (rho = 0.542, p < 0.001), LAG3 (rho = 0.536, p < 0.001), CTLA4 (rho = 0.496, p < 0.001), and TIGIT (rho = 0.419, p < 0.001) (Fig. 4G). Moreover, chemokines and chemokine receptors were also reported to play crucial roles in the infiltration of immune cells into tumors [43]. Therefore, we also analyzed the correlation between TCIRG1 expression and immune cell chemokines and chemokine receptors using the TISIDB database. The heatmap results showed that TCIRG1 was significantly positively correlated with the expression of some chemokines and chemokine receptors in KIRC (Supplementary Figure S2G, I), including CCL5 (rho = 0.497, p < 0.001), XCL2 (rho = 0.457, p < 0.001), CXCL13 (rho = 0.444, p < 0.001), XCL1 (rho = 0.412, p < 0.001), CXCR3 (rho = 0.497, p < 0.001), CXCR5 (rho = 0.41, p < 0.001), CCR10 (rho = 0.355, p < 0.001), and CCR5 (rho = 0.31, p < 0.001) (Supplementary Figures S2H, J). These results suggest that TCIRG1 may play an essential role in regulating tumor immunity.

### Downregulation of TCIRG1 expression inhibits the proliferation, migration and invasion of ccRCC cell lines and induces their apoptosis

**Fig. 5:**
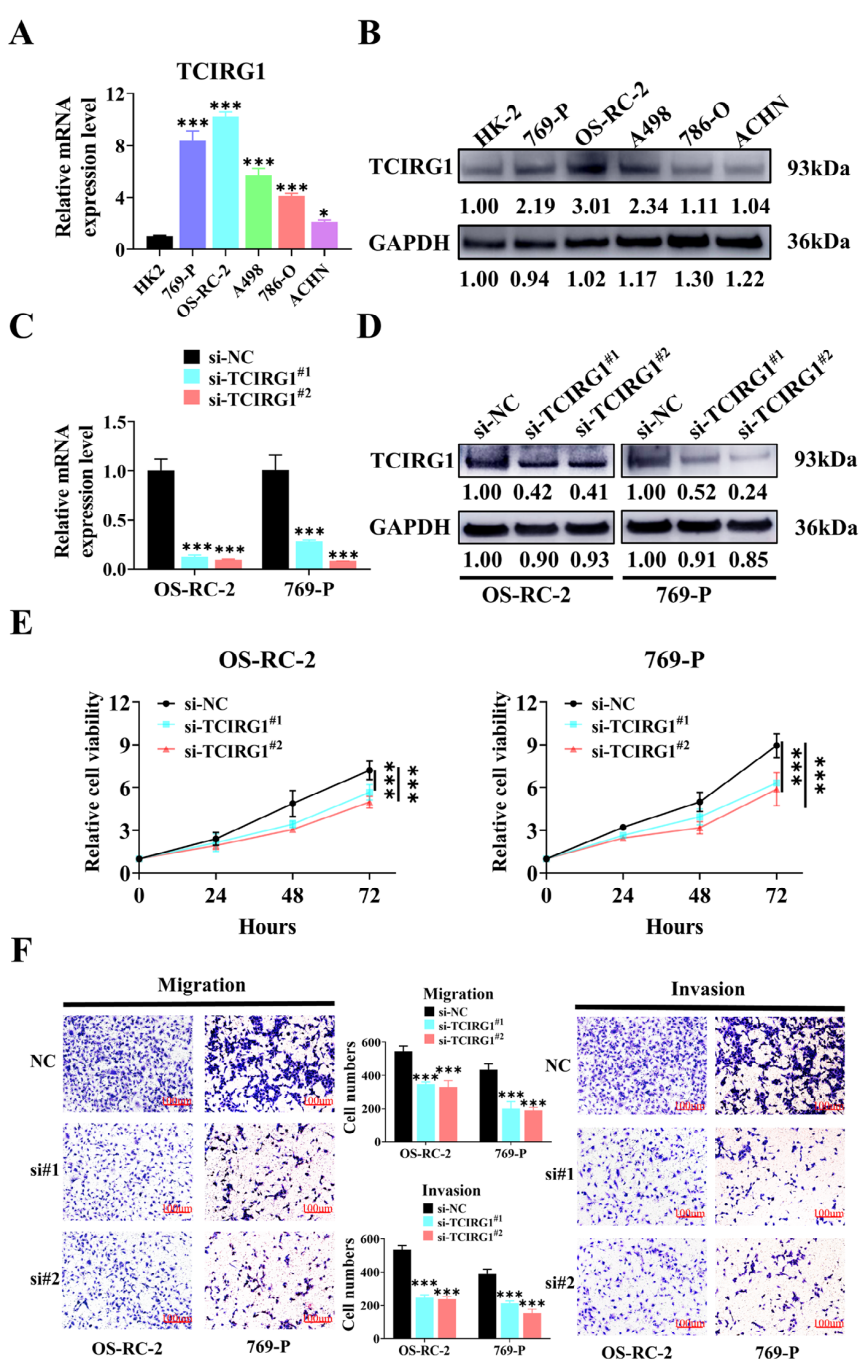
Downregulation of TCIRG1 expression inhibits the proliferation, migration and invasion of ccRCC cell lines. (**A**-**B**) RT-qPCR results of TCIRG1 (**A**) and representative Western blot images with relative gray values and histograms (**B**) for TCIRG1 mRNA and protein in different ccRCC cell lines (769-P, 786-O, OS-RC-2, A498, ACHN), using normal human renal cell line (HK-2) as normal controls. (**C**-**D**) RT-qPCR results of TCIRG1 (**C**) and representative Western blot images with relative gray values and histograms (**D**) showed that siRNA silenced the mRNA and protein expression of TCIRG1 in OS-RC-2 or 769P, respectively. (**E**) CCK-8 was used to detect the proliferation of OS-RC-2 or 769P cells during TCIRG1 knockdown. The proliferation rate showed a multiple change relative to the control group. (**F**) In the renal cell carcinoma cell lines (OS-RC-2 and 769-P) transfected with TCIRG1 (control and si-TCIRG1), the migration ability was detected by Transwell, and the invasion ability was detected by matrigel. (All p values are defined as: *p < 0.05, **p < 0.01 and ***p < 0.001). ccRCC, clear cell renal cell carcinoma; RT-qPCR, real-time polymerase chain reaction; siRNA, Small interfering RNA; CCK‐8, Cell Counting Kit‐8; FITC, fluorescein isothiocyanate; PI, propidium iodide

**Fig. 6:**
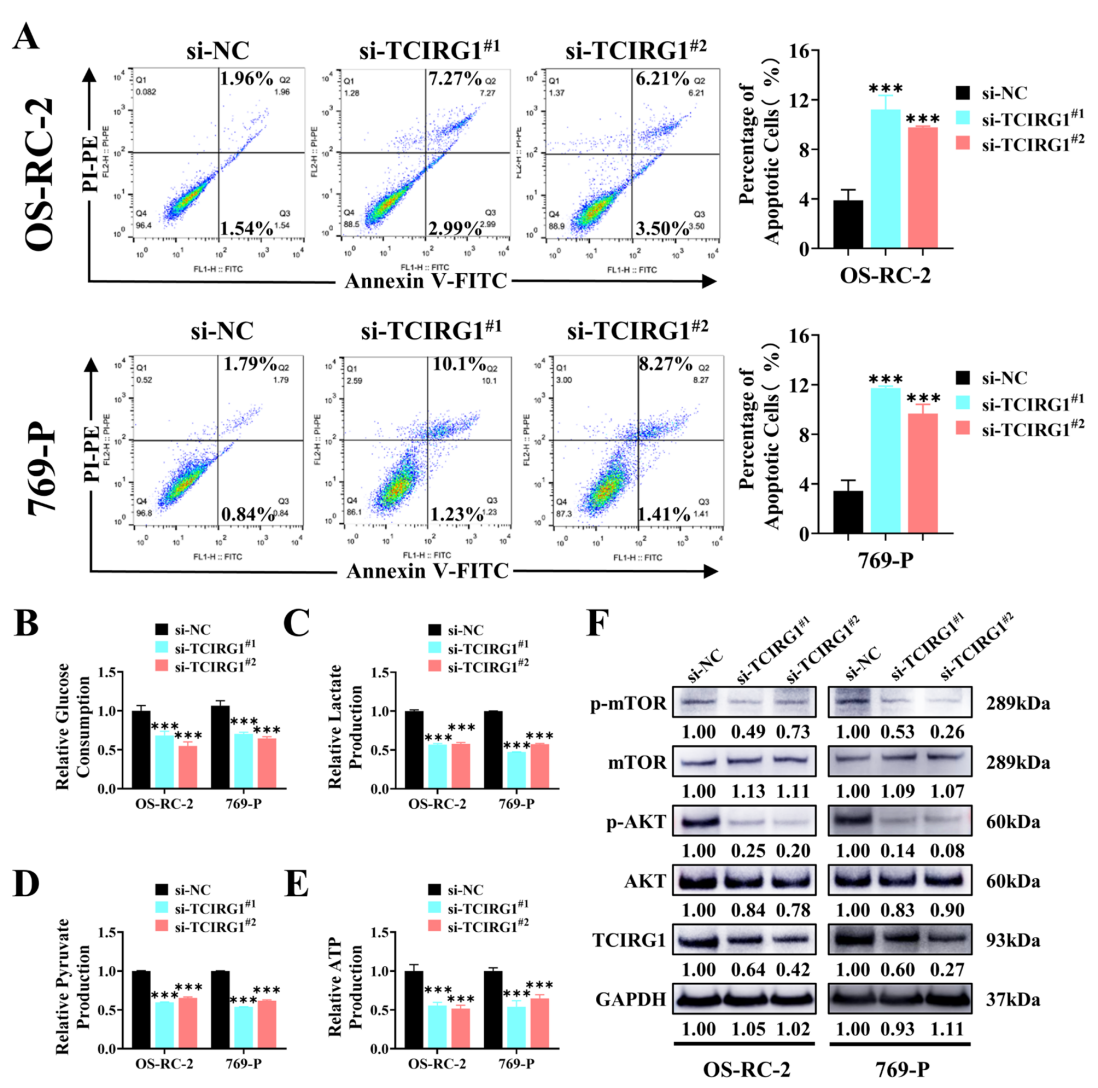
Downregulation of TCIRG1 expression inhibits migration, invasion, glycolysis processes, and the AKT/mTOR signaling pathway in the ccRCC cell lines. (**A**) Annexin V-FITC/PI double-staining of OS-RC-2 or 769-P cells with or without TCIRG1 knockdown was performed, and flow cytometry assays were employed to detect the percentage of apoptotic cells. (**B**-**E**) Glucose consumption (**B**), lactate production (**C**), pyruvate production (**D**), and intracellular ATP production (**E**) were detected in TCIRG1 transfected (control and si-TCIRG1) renal cell carcinoma cell lines (OS-RC-2 and 769-P). (**F**) The expression level of AKT/mTOR signaling pathway related proteins in renal cell carcinoma cell lines (OS-RC-2 and 769-P) transfected with TCIRG1 (control and si-TCIRG1) was detected. (All p values are defined as: *p < 0.05, **p < 0.01 and ***p < 0.001). ccRCC, clear cell renal cell carcinoma; mTOR, Mechanistic Target of Rapamycin; ATP, Association of Tennis Professionals

Based on the previous finding in the online database that the high TCIRG1 expression was associated with poor prognosis in KIRC patients, we conducted in vitro experiments to further investigate the effect of TCIRG1 expression in ccRCC cell lines. First, we investigated the expression of TCIRG1 in common ccRCC cell lines 769-P, 786-O, OS-RC-2, A498 and ACHN, using renal tubular epithelial cell line HK-2 as a control group. The results of RT-qPCR and western blot showed that the expression of TCIRG1 in ccRCC cell lines was higher than that of HK-2, especially in OS-RC-2 and 769-P cell lines (Fig. 5A, B). To determine whether TCIRG1 affected the growth of ccRCC cell lines, we used small interfering RNA (siRNA) to knockdown TCIRG1 in OS-RC-2 and 769-P cells, screened out si-TCIRG1^#1^ and si-TCIRG1^#2^ through RT-qPCR (Supplementary Figure S2K), and verified the knockdown efficiency using Western blot (Fig. 5C, D). Then, the proliferation ability of ccRCC cell lines (OS-RC-2 and 769-P) was measured by cell counting kit 8 (CCK-8) proliferation experiment. The results showed that downregulation of TCIRG1 (si-TCIRG1^#1^, si-TCIRG1^#2^) inhibited the proliferation of ccRCC cells (Fig. 5E). We then used transwell and matrix gel analysis to detect the effect of TCIRG1 on the migration and invasion of OS-RC-2 and 769-P cells. Compared with the control group, TCIRG1 knockdown decreased the migration and invasion of OS-RC-2 and 769-P cells markedly (Fig. 5F). Subsequent flow cytometry analysis showed that TCIRG1 knockdown increased the apoptosis of ccRCC cells as compared with the corresponding control cells (Fig. 6A). In summary, the results show that downregulating the expression of TCIRG1 inhibited the proliferation, migration and invasion of ccRCC cell lines and promoted their apoptosis, suggesting that TCIRG1 played an extremely critical role in the growth and development of ccRCC cell lines.

### Downregulation of TCIRG1 inhibits glycolysis and AKT/mTOR signaling pathway in ccRCC cell lines

TCIRG1 is a biomarker selected from the set of genes associated with glycolysis. To investigate the effect of TCIRG1 on glycolytic process in ccRCC cell lines, we measured glucose uptake, lactate production, pyruvate production, and ATP production in ccRCC cell lines after TCIRG1 knockdown. As shown in Fig. 6B-E, downregulation of TCIRG1 decreased glucose uptake, lactate production, pyruvate production, and ATP production, suggesting that downregulation of TCIRG1 could inhibit glycolysis in ccRCC cell lines. To examine the effect of the glycolysis-related gene TCIRG1 on the AKT/mTOR signaling pathway, we performed western blot analysis and found that down-regulation of TCIRG1 reduced phosphorylated AKT (p-AKT) and phosphorylated mTOR (p-mTOR) (Fig. 6F). All these results suggest that downregulation of TCIRG1 could inhibit aerobic glycolysis of ccRCC through the AKT/mTOR signaling pathway.

## Discussion

Tumor cells are metabolically reprogrammed to promote their own growth, metastasis and survival. Decades of genomic research on RCC have shown that RCC is a hypermetabolic disease [45]. The histology of RCC shows that metabolic activity increases with disease progression, especially aerobic glycolysis [46]. Differential regulation of glycolysis between tumor and immune cells provides an opportunity for selective inhibition of glucose metabolism in tumors and a unique window in the search for more effective cancer immunotherapies [47]. Tumor cells exhibit increased glycolytic dependence by increasing glucose uptake and glucose fermentation into lactate to meet the high anabolic demand for tumor cell proliferation [14]. Study has shown that metabolic interventions can significantly improve the efficacy of immunotherapy [48, 49]. Thus, the combination of immunotherapy and metabolic interventions is a promising strategy for improving therapeutic outcomes [50]. To improve the efficacy of immune checkpoint therapies in RCC, we identified TCIRG1, a biomarker that regulates aerobic glycolysis in ccRCC.

Many studies have demonstrated that increased glucose metabolism caused by glycolysis can promote the growth, proliferation and long-term maintenance of tumor cells, which is an important marker of malignant progression of cancer [51]. Downregulation of SPTBN1 was found to promote the progression of ccRCC by activating GPT2-dependent aerobic glycolysis [52]. TCIRG1 was first identified in osteosclerosis, and study has shown that TCIRG1 mutation is a common cause of human autosomal recessive osteosclerosis [22]. TCIRG1 acts as a metastasis enhancer by regulating growth and EMT in HCC cells [24]. In glioblastoma multiforme (GBM), TCIRG1 is considered as a prognostic biomarker and an indicator of immune infiltration [53].

To the best of our knowledge, there is no study on TCIRG1 and aerobic glycolysis in renal cancer. It was found in our study that TCIRG1 was biomarker associated with glycolysis and an independent prognostic risk factor for ccRCC. In addition, high expression of TCIRG1 was associated with malignancy progression and poor prognosis in ccRCC patients, and positively correlated with the immunoinhibitors PDCD1 and CTLA4 (Fig. 4G), and IFN-γ as well (Fig. 2A). PDCD1 and CTLA4 are two key T cell immune checkpoint molecules, which can negatively regulate T cell glycolysis and mitochondrial metabolism [54], and PD-1 blockage can restore aerobic glycolysis and IFNγ production in T cells [55]. As described in our previous findings, down-regulation of TCIRG1 could inhibit aerobic glycolysis in ccRCC cell lines (Fig. 6B-F). In acute myeloid leukemia, high expression of PD-L1 was found to promote aerobic glycolysis via the Akt/mTOR/HIF-1α axis [56]. In ccRCC cell lines, knockdown PBRM1 was found to activate the AKT/mTOR signaling pathway and increase the expression of key glycolytic enzymes at mRNA and protein levels [57]. Therefore, we speculate that TCIRG1 may inhibit aerobic glycolysis in ccRCC, thereby regulating the malignant progression of ccRCC. This speculation may provide a new idea for the treatment of ccRCC by combining metabolic intervention with immunotherapy.

It is for the first time that we identified TCIRG1 as a potential biomarker of aerobic glycolysis in ccRCC cell lines and found that TCIRG1 could regulate aerobic glycolysis in ccRCC, thereby modulating its malignancy progression. In addition, we also explored the diversity of TCIRG1 and immune cell infiltration, as well as the relation of TCIRG1 with immunotherapy by using various bioinformatics methods, including clinical information analysis, GSEA enrichment analysis, immune infiltration analysis, and multi-omics data.

However, the study has some limitations. First, our study did not elucidate the specific mechanism by which TCIRG1 affected aerobic glycolysis in ccRCC cell lines through the AKT/mTOR signaling pathway. In addition, the enrolled patient information and multiple data sets that we analyzed should be derived from a real-world cohort to more accurately validate the prognostic impact of biomarkers on the response to immunotherapy.

## Conclusion

In this study, we identified a glycolysis-related biomarker TCIRG1 in ccRCC and found that the expression of TCIRG1 was closely related to immune response and immunotherapy. Our data have shown that high expression of TCIRG1 predicts malignancy progression and poor prognosis for ccRCC. We found that TCIRG1 knockdown inhibited the proliferation, migration and invasion of ccRCC cell lines and promoted cell apoptosis, suggesting that TCIRG1 may regulate aerobic glycolysis and malignant progression of ccRCC. These findings reveal the relationship between aerobic glycolysis and immunotherapy in ccRCC, thus providing a novel direction for the treatment of ccRCC by combination of metabolic intervention and immunotherapy.

### Electronic supplementary material

Below is the link to the electronic supplementary material.

Supplementary Material 1

Supplementary Material 2

Supplementary Material 3

Supplementary Material 4

Supplementary Material 5

Supplementary Material 6

## Data Availability

The data and material during the current study were available from the corresponding author on reasonable request.

## Abbreviations

ccRCC: Clear cell renal cell carcinoma
DEGs: Differentially expressed genes
OS: Overall survival
PFI: Progression-free interval
RCC: Renal cell carcinoma
TCIRG1: T-cell immune regulator 1
TME: Tumor microenvironment
