# Supplementary figures and images for: Glycolysis-related biomarker TCIRG1 participates in regulation of renal cell carcinoma progression and tumor immune microenvironment by affecting aerobic glycolysis and AKT/mTOR signaling pathway
Source: Cancer Cell Int. 2023 Aug 30;23:186. doi: 10.1186/s12935-023-03019-0 (PMC10468907; doi:10.1186/s12935-023-03019-0)

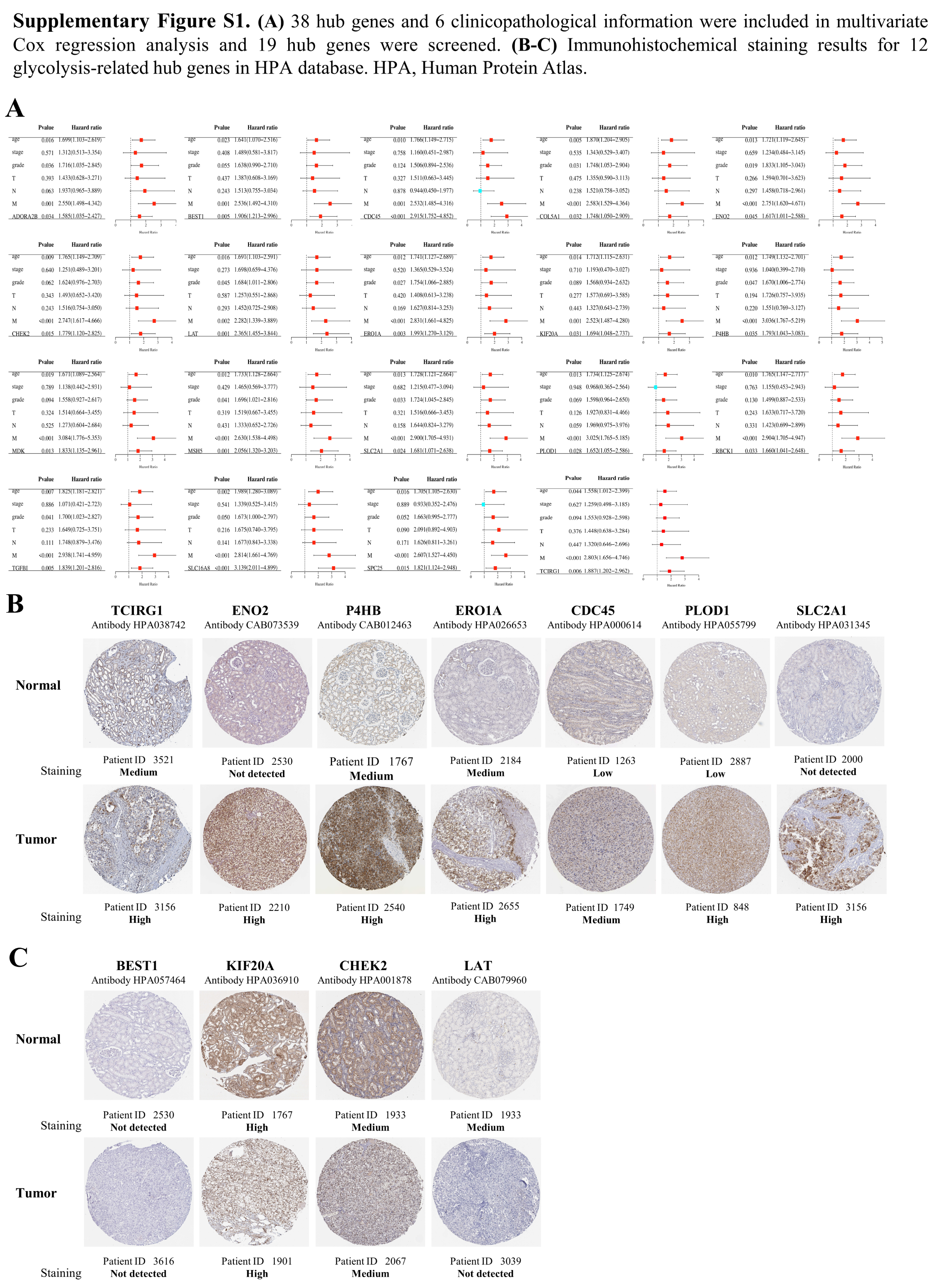

Supplement: Supplementary file 1 — Supplementary Material 1 [file 12935_2023_3019_MOESM1_ESM.tif]

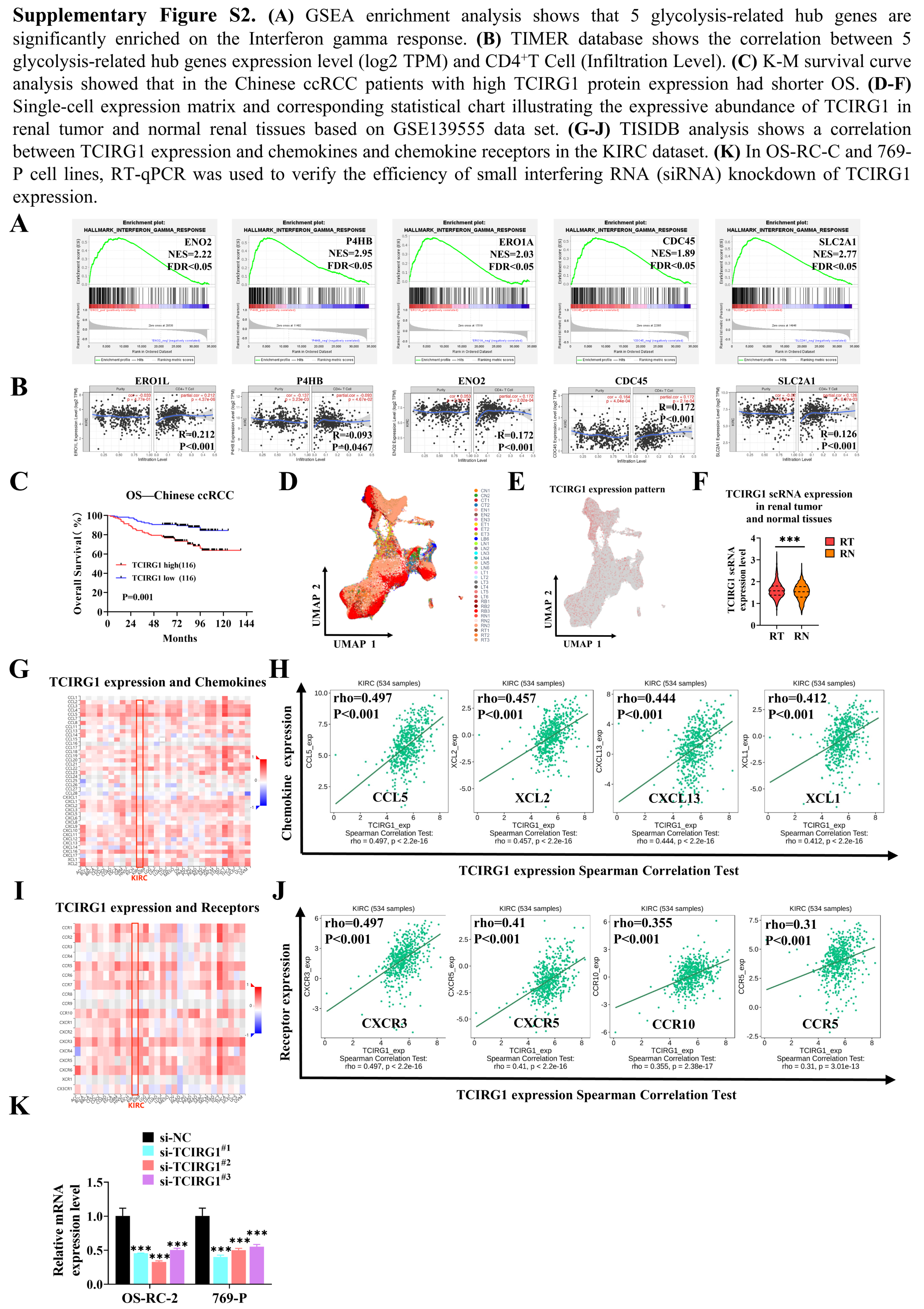

Supplement: Supplementary file 2 — Supplementary Material 2 [file 12935_2023_3019_MOESM2_ESM.tif]
