# Supplementary material for: Glycolysis-related biomarker TCIRG1 participates in regulation of renal cell carcinoma progression and tumor immune microenvironment by affecting aerobic glycolysis and AKT/mTOR signaling pathway
Source: Cancer Cell Int. 2023 Aug 30;23:186. doi: 10.1186/s12935-023-03019-0 (PMC10468907; doi:10.1186/s12935-023-03019-0)
Supplement: Supplementary file 5 — Supplementary Material 5 [file 12935_2023_3019_MOESM5_ESM.docx]

**Supplementary Table S1:** Clinicopathologic characteristics of ccRCC patients by TCIRG1 expression in combined cohort (n = 525)

|  | **TCIRG1 Expression*** | |  |  |
| --- | --- | --- | --- | --- |
|  | **TCIRG1^low^** | **TCIRG1^high^** | **Sum** |  |
| **Characteristics** | **(n=330)** | **(n=195)** | **(525)** | **p Value** |
| Age |  |  |  | 0.928 |
| ＜60 | 158 | 92 | 250 |  |
| ≥60 | 172 | 103 | 275 |  |
| Gender |  |  |  | ＜0.001 |
| Male | 136 | 132 | 268 |  |
| Female | 194 | 63 | 257 |  |
| TNM stage |  |  |  | ＜0.001 |
| I-II | 230 | 88 | 318 |  |
| III-IV | 96 | 107 | 203 |  |
| NA | 4 |  | 4 |  |
| Pathological grade |  |  |  | ＜0.001 |
| 1-2 | 186 | 56 | 242 |  |
| 3-4 | 140 | 137 | 277 |  |
| NA | 4 | 2 | 6 |  |
| Overall survival |  |  |  | ＜0.001 |
| Alive | 246 | 99 | 345 |  |
| Dead | 84 | 96 | 180 |  |
| Progression Free Interval |  |  |  | 0.026 |
| Free of progression | 236 | 121 | 357 |  |
| Progressed | 94 | 74 | 168 |  |

* TCIRG1 was divided into high and low expression groups with an optimal cutoff value of 3.82

TCIRG1 low expression: Gene expression level of TCIRG1<3.82;

TCIRG1 high expression: Gene expression level of TCIRG1≥3.82;

Statistical significance was calculated by Chi squared test or Fisher’s exact test for categorical/binary measures.

Abbreviation: ccRCC, clear cell renal cell carcinoma.

**Supplementary Table S2:** Clinicopathologic characteristics of ccRCC patients by TCIRG1 expression in training cohort (n=262).

| **Characteristics** | **TCIRG1 Expression*** | |  |  |
| --- | --- | --- | --- | --- |
|  | **TCIRG1^low^** | **TCIRG1^high^** | **Sum** |  |
|  | **(n=162)** | **(n=100)** | **(262)** | **p Value** |
| Age |  |  |  | 0.899 |
| ＜60 | 73 | 46 | 119 |  |
| ≥60 | 89 | 54 | 143 |  |
| Gender |  |  |  | 0.682 |
| Male | 113 | 67 | 180 |  |
| Female | 49 | 33 | 82 |  |
| TNM stage |  |  |  | 0.004 |
| I-II | 110 | 48 | 158 |  |
| III-IV | 52 | 50 | 102 |  |
| NA | 0 | 2 | 2 |  |
| Pathological grade |  |  |  | 0.015 |
| 1-2 | 82 | 35 | 117 |  |
| 3-4 | 77 | 63 | 140 |  |
| NA | 3 | 2 | 5 |  |
| Overall survival |  |  |  | 0.001 |
| Alive | 124 | 57 | 181 |  |
| Dead | 38 | 43 | 81 |  |
| Progression Free Interval |  |  |  | 0.008 |
| Free of progression | 125 | 61 | 186 |  |
| Progressed | 37 | 39 | 76 |  |

* TCIRG1 was divided into high and low expression groups with an optimal cutoff value of 3.82

TCIRG1 low expression: Gene expression level of TCIRG1<3.82;

TCIRG1 high expression: Gene expression level of TCIRG1≥3.82;

Statistical significance was calculated by Chi squared test or Fisher’s exact test for categorical/binary measures.

Abbreviation: ccRCC, clear cell renal cell carcinoma.

**Supplementary Table S3:** Clinicopathologic characteristics of ccRCC patients by TCIRG1 expression in validation cohort (n=263).

| **Characteristics** | **TCIRG1 Expression*** | |  |  |
| --- | --- | --- | --- | --- |
|  | **TCIRG1^low^** | **TCIRG1^high^** | **Sum** |  |
|  | **(n=165)** | **(n=98)** | **(263)** | **p Value** |
| Age |  |  |  | 0.899 |
| ＜60 | 79 | 46 | 125 |  |
| ≥60 | 86 | 52 | 138 |  |
| Gender |  |  |  | 0.190 |
| Male | 97 | 66 | 163 |  |
| Female | 68 | 32 | 100 |  |
| TNM stage |  |  |  | <0.001 |
| I-II | 115 | 44 | 159 |  |
| III-IV | 49 | 54 | 103 |  |
| NA | 1 |  | 1 |  |
| Pathological grade |  |  |  | <0.001 |
| 1-2 | 93 | 28 | 121 |  |
| 3-4 | 70 | 69 | 139 |  |
| NA | 2 | 1 | 3 |  |
| Overall survival |  |  |  | <0.001 |
| Alive | 123 | 50 | 173 |  |
| Dead | 42 | 48 | 90 |  |
| Progression Free Interval |  |  |  | 0.133 |
| Free of progression | 118 | 61 | 179 |  |
| Progressed | 47 | 37 | 84 |  |

* TCIRG1 was divided into high and low expression groups with an optimal cutoff value of 3.82

TCIRG1 low expression: Gene expression level of TCIRG1<3.82;

TCIRG1 high expression: Gene expression level of TCIRG1≥3.82;

Statistical significance was calculated by Chi squared test or Fisher’s exact test for categorical/binary measures.

Abbreviation: ccRCC, clear cell renal cell carcinoma.

**Supplementary Table S4:** Univariate and multivariate Cox regression analysis of TCIRG1 expression classifier and clinical characteristics with Overall Survival and Progression Free Survival in combined cohort (n=525).

| **Characteristics** | **Overall survival** |  |  |  |  |  | **Progression Free Interval** | | |  | | |  | | | |  | |  |
| --- | --- | --- | --- | --- | --- | --- | --- | --- | --- | --- | --- | --- | --- | --- | --- | --- | --- | --- | --- |
|  | **Univariate** |  |  | **Multivariate** |  |  | **Univariate** |  | | |  | | | **Multivariate** | | | |  | |
|  | **HR (95%CI)** | **p Value** |  | **HR (95%CI)** | **p Value** |  | **HR (95%CI)** | **p Value** | | |  | | | **HR (95%CI)** | **p Value** | | | | |
| Age  (≥60 vs<60) | 1.740(1.282-2.361) | <0.001 |  | 1.424(1.041-1.949) | 0.027 |  | 1.388(1.020-1.889) | 0.037 |  | | | 0.982(0.713-1.351) | | | | 0.910 | | | |
| Gender  (Male vs Female) | 0.810(0.602-1.089) | 0.163 |  |  |  |  | 1.255(0.906-1.738) | 0.172 |  | | |  | | | |  | | | |
| TNM stage  (III-IV vs I-II) | 4.811(3.494-6.624) | <0.001 |  | 3.534(2.509-4.976) | <0.001 |  | 7.124(5.007-10.134) | <0.001 |  | | | 5.938(4.075-8.652) | | | | <0.001 | | | |
| Pathological grade  (III-IV vs I-II) | 2.483(1.791-3.442) | <0.001 |  | 1.444(1.023-2.039) | 0.037 |  | 3.083(2.171-4.378) | <0.001 |  | | | 2.009(1.379-2.926) | | | | <0.001 | | | |
| TCIRG1 expression  (High vs Low) | 2.398(1.787-3.217) | <0.001 |  | 1.716(1.261-2.334) | 0.001 |  | 1.675(1.233-2.277) | 0.001 |  | | | 0.912(0.657-1.265) | | | | 0.580 | | | |

* TCIRG1 was divided into high and low expression groups with an optimal cutoff value of 3.82

TCIRG1 low expression: Gene expression level of TCIRG1<3.82;

TCIRG1 high expression: Gene expression level of TCIRG1≥3.82;

Statistical significance was calculated by Chi squared test or Fisher’s exact test for categorical/binary measures.

Abbreviation: ccRCC, clear cell renal cell carcinoma.
